# Characterizing the Mechanical Properties of Running-Specific Prostheses

**DOI:** 10.1371/journal.pone.0168298

**Published:** 2016-12-14

**Authors:** Owen N. Beck, Paolo Taboga, Alena M. Grabowski

**Affiliations:** 1 Department of Integrative Physiology, University of Colorado, Boulder, Colorado, United States of America; 2 Department of Veterans Affairs, Eastern Colorado Healthcare System, Denver, Colorado, United States of America; Northwestern University, UNITED STATES

## Abstract

The mechanical stiffness of running-specific prostheses likely affects the functional abilities of athletes with leg amputations. However, each prosthetic manufacturer recommends prostheses based on subjective stiffness categories rather than performance based metrics. The actual mechanical stiffness values of running-specific prostheses (i.e. kN/m) are unknown. Consequently, we sought to characterize and disseminate the stiffness values of running-specific prostheses so that researchers, clinicians, and athletes can objectively evaluate prosthetic function. We characterized the stiffness values of 55 running-specific prostheses across various models, stiffness categories, and heights using forces and angles representative of those measured from athletes with transtibial amputations during running. Characterizing prosthetic force-displacement profiles with a 2^nd^ degree polynomial explained 4.4% more of the variance than a linear function (p<0.001). The prosthetic stiffness values of manufacturer recommended stiffness categories varied between prosthetic models (p<0.001). Also, prosthetic stiffness was 10% to 39% less at angles typical of running 3 m/s and 6 m/s (10°-25°) compared to neutral (0°) (p<0.001). Furthermore, prosthetic stiffness was inversely related to height in J-shaped (p<0.001), but not C-shaped, prostheses. Running-specific prostheses should be tested under the demands of the respective activity in order to derive relevant characterizations of stiffness and function. In all, our results indicate that when athletes with leg amputations alter prosthetic model, height, and/or sagittal plane alignment, their prosthetic stiffness profiles also change; therefore variations in comfort, performance, etc. may be indirectly due to altered stiffness.

## Introduction

Running is a bouncing gait that is well-characterized by a spring-mass model [1–3]. The spring-mass model portrays the stance leg as a mass-less linear spring supporting a point mass representing the runner’s center of mass. Upon ground contact, the leg spring compresses and stores elastic energy until mid-stance, and then returns mechanical energy from mid-stance through the end of ground contact [4]. In this model, the leg spring is completely elastic, however the structures of a biological leg are viscoelastic and therefore only a portion of the stored potential elastic energy is returned (due to hysteresis). The spring-like action of the leg conserves a portion of the runner’s mechanical energy, theoretically mitigating the additional muscular force and mechanical energy input necessary to maintain running speed [4,5]. The magnitude of the stored and returned mechanical energy is inversely related to leg stiffness (resistance to compression), and is influenced by the magnitude and orientation of the external force vector acting on the leg [1]. Simply modeled as a linear spring, leg stiffness (*k*_*leg*_) equals the quotient of the peak applied force (*F*) and the change in leg length (Δ*l*) from touchdown to mid-stance [2]:

$$k_{leg}=\frac{F}{\Delta l}$$ (1)

Inspired by the spring-like nature of running, passive-elastic running-specific prostheses (RSPs) were developed to enable athletes with lower-limb amputations to run. These carbon-fiber devices are attached to the sockets that encompass the residual limbs, are in-series with the residual limbs, and mimic the mechanical energy storage and return of tendons during ground contact. Unlike biological ankles, RSPs cannot generate mechanical power anew and only return 63% to 95% of the stored elastic energy during running [6–8]. For context, biological ankles generate mechanical power through use of elastic structures as well as muscles, and thus appear to “return” 241% of the energy stored while running at 2.8 m/s [7].

Athletes with leg amputations may adapt similar leg spring mechanics as non-amputees by using RSPs that emulate biological lower leg stiffness. Individually, non-amputees adopt a constant [2,9,10], metabolically optimal leg stiffness during running [11–13]. Non-amputee runners maintain leg stiffness across speeds by exhibiting constant ankle joint stiffness (sagittal plane torsional stiffness) [14,15]. It has been assumed that prosthetic stiffness is also constant across speeds [8,16–18], which if true, RSPs would act like that of biological ankles [14,15]. Yet, McGowan et al. [16] reported that the affected leg stiffness of athletes with transtibial amputations decreases as speed increases from 3.0 m/s to top speed (the range of top speeds achieved were 7.0 m/s to 10.8 m/s), indicating that prosthetic stiffness and/or affected leg knee stiffness may be inversely related with speed. Moreover, Dyer et al. [19] mechanically tested two Elite Blade RSPs (Chas A Blatchford & Sons Ltd. Basingstoke, UK) in a materials testing machine and reported that the RSPs have curvilinear force-displacement profiles, suggesting that prosthetic stiffness is non-constant and force dependent. Due to conflicting evidence in the literature, coupled with insufficient information provided by manufacturers regarding prosthetic stiffness profiles, it is unknown whether the force-displacement profiles of RSPs are linear, or curvilinear, which would infer that stiffness is contingent upon the applied force magnitude.

Prosthetic manufacturers do not report the stiffness values of RSPs (e.g. in kN/m). Instead, they classify RSPs into predetermined stiffness categories (e.g. categories 1 to 7), which are recommended to users based on body mass and intended activity (slow or fast running) [20–22]. Larger/heavier athletes with amputations are generally prescribed RSPs with numerically greater stiffness categories, which are presumably stiffer than numerically lower stiffness categories. Additionally, some prosthetic models are recommend at greater stiffness categories for fast running than for slow running [20,21], whereas other models are recommended at the same stiffness category irrespective of intended running speed [23,24]. These inconsistencies in prosthetic stiffness recommendations persist despite the potential influence of stiffness on running mechanics and performance. Therefore, it is imperative to quantify and disseminate stiffness values to further understand prosthetic function.

To accurately quantify prosthetic stiffness, it seems obvious to evaluate RSPs using forces and angles indicative of those produced during the respective activity. When athletes with transtibial amputations run, they generate peak vertical ground reaction forces (GRFs) with their affected legs that are 2.1 to 3.3 times body weight at speeds of 2.5 m/s to 10.8 m/s [8,18,25,26]. During running, peak resultant GRFs typically occur around mid-stance and are oriented vertically. At the same instant, the proximal end of the stance leg’s RSP is rotated forward in the sagittal plane relative to the peak resultant GRF vector. Therefore, the proximal bending moment acting on shorter RSPs may be less than that on taller RSPs for a given applied force, due to a reduced moment arm length. A smaller moment (torque) associated with shorter RSPs may reduce vertical displacement, and in turn increase prosthetic stiffness. Nonetheless, the peak resultant GRF magnitudes and sagittal plane orientations relative to RSPs are unknown, as is the influence of prosthetic height on stiffness.

Since prosthetic stiffness and hysteresis likely affect running performance, we aimed to 1) characterize the force-displacement profiles of RSPs, 2) quantify and compare prosthetic stiffness and 3) hysteresis values across prosthetic models, stiffness categories, and heights using angles and forces that replicate those exhibited during running, and 4) determine whether prosthetic height affects stiffness. Such information will enable accurate and objective comparisons between RSPs, subsequently allowing for potential improvements in prosthetic design, prescription, and athletic performance. Based on the predominant assumption that prosthetic stiffness is constant during running [8,16–18]; we hypothesized that the force-displacement profiles of RSPs would be linear. We hypothesized that for a given body mass and running speed, manufacturer recommended prosthetic stiffness would be similar between models. We also hypothesized that the magnitude of prosthetic hysteresis would not differ across testing conditions. Lastly, we hypothesized that shorter RSPs would be stiffer than taller RSPs.

## Methods

### Testing Procedure

We measured GRFs and sagittal plane angles of RSPs relative to the peak resultant GRFs from 11 athletes (5 males and 6 females; mean ± SD; age: 27.8 ± 5.7; standing height: 1.74 ± 0.08 m; body mass: 68.9 ± 15.3 kg) with unilateral transtibial amputations while they ran at 3 m/s and 6 m/s on a force-measuring treadmill. Each athlete used their own personal RSP. 3 m/s represents a typical distance running speed [27–29] and 6 m/s represents the fastest speed that all of our participants could achieve. The Intermountain Healthcare IRB, Colorado Multiple IRB, and the USAMRMC Office of Research Protection, Human Research Protection Office approved this study. Prior to participating, nine athletes provided informed written consent in accordance with the Intermountain Healthcare IRB and two participants provided informed written consent in accordance with the Colorado Multiple IRB and USAMRMC Office of Research Protection, Human Research Protection Office. Data collection took place in two separate labs.

**Fig 1:**
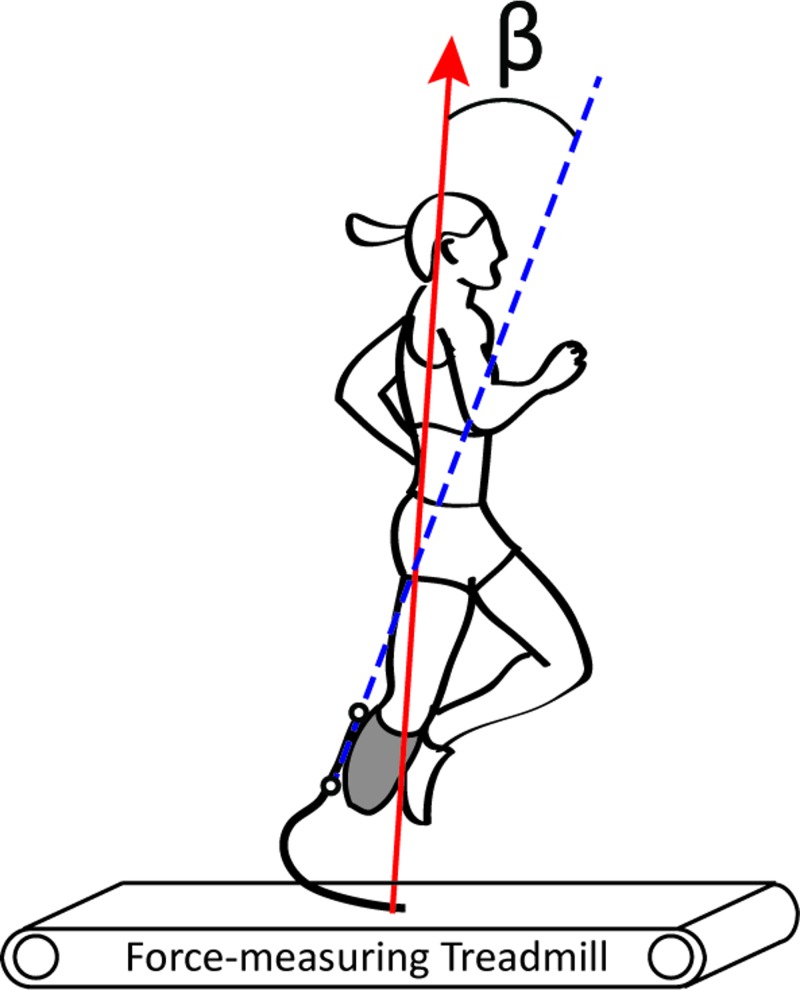
Biomechanics of running. Illustration of the calculated angle (β) between the longitudinal axis of the running-specific prosthesis (dashed blue line) and the peak resultant GRF vector (solid red arrow).

We placed reflective markers on the lateral proximal and distal ends of each RSP’s longitudinal axis and measured segment motion during each trial using a motion capture system (Motion Analysis Corporation, Santa Rosa, CA, USA, or Vicon Nexus, Oxford, UK) at 240 Hz (lab 1) or 200 Hz (lab 2) and implemented a 4^th^ order low-pass Butterworth filter with a cutoff frequency of 6 Hz (Visual 3D, C-motion, Inc., Germantown, MD, USA) (Fig 1). The longitudinal axis was defined by a line through the center of the pylon connecting each socket to the corresponding C-shaped RSP, and along the center of the proximal, longitudinal section of each J-shaped RSP (Fig 1). Four athletes used a C-shaped RSP, and seven used a J-shaped RSP. We recorded GRFs via force-measuring treadmills (Treadmetrix, Park City, UT, USA) at 2400 Hz (lab 1) or 1000 Hz (lab 2) and applied a 4^th^ order low-pass Butterworth filter with a cutoff frequency of 30 Hz using a custom MATLAB script (MathWorks Inc, Natick, MA, USA). Our data were comparable because each participant ran both speeds at one lab, and due to the implementation of the same filtering process.

We determined the peak GRF magnitude, as well as the average sagittal plane angle of the longitudinal axis for each athlete’s RSP relative to the peak resultant GRF vector from 10 consecutive ground contacts with the affected leg. We assessed the average angles for trials performed with C-shaped RSPs at 3 m/s (α_3_) and 6 m/s (α_6_), and with J-shaped RSPs at 3 m/s (β_3_) and 6 m/s (β_6_). When the RSP’s longitudinal axis is parallel to the peak resultant GRF vector, the RSP is at 0°. Positive angles indicate that the proximal longitudinal axis was rotated forward in the sagittal plane relative to the peak resultant GRF vector (Fig 1). Sequentially, we implemented the measured angles (α_3_, α_6_, β_3,_ and β_6_) and peak resultant GRF magnitudes into our prosthetic testing procedure.

### Running-Specific Prostheses

**Fig 2:**
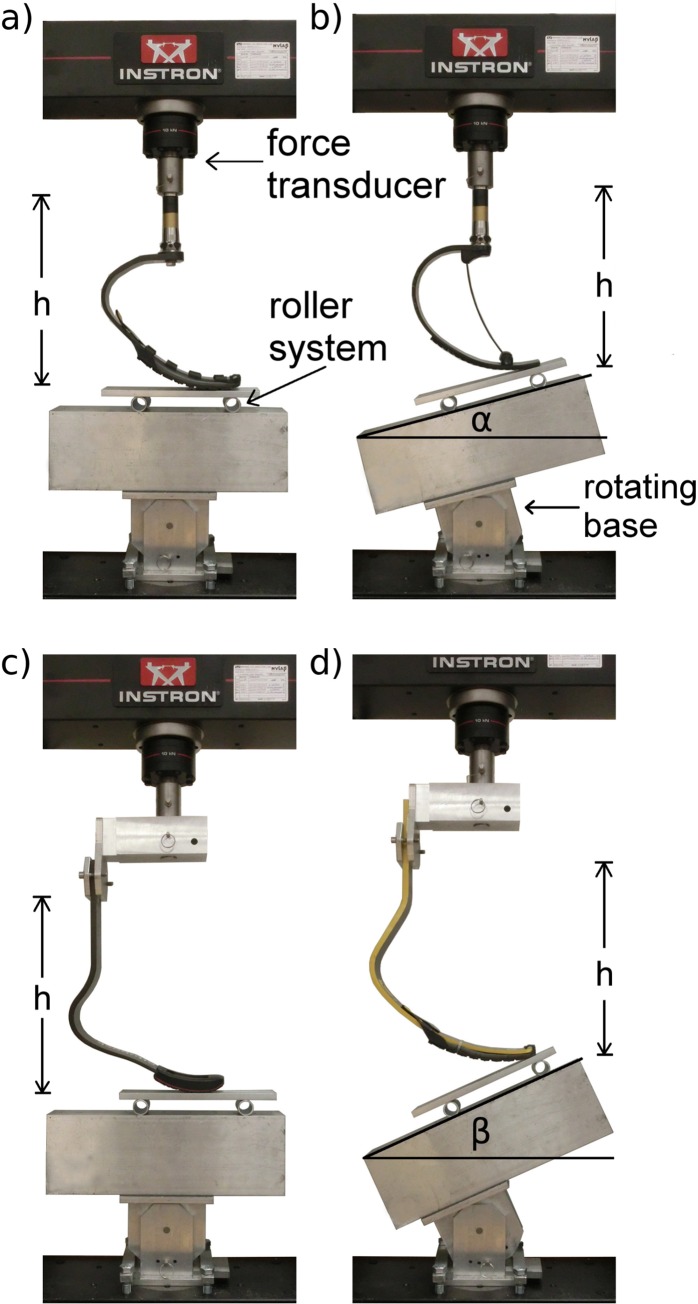
Material testing setup with each running specific-prosthetic model. Each running specific-prosthesis (RSP) was tested with the respective manufacturer’s rubber sole (Össur Cheetah Xtend prosthesis was equipped with an Össur Flex-Run’s sole), using our rotating base, and low-friction roller system. a) An Össur Flex-Run prosthesis (C-shaped) tested at 0°. b) A Freedom Innovations Catapult prosthesis (C-shaped) tested at α° (3 m/s). c) An Ottobock 1E90 Sprinter prosthesis (J-shaped) tested at neutral (0°). d) An Össur Cheetah Xtend prosthesis (J-shaped) tested at β° (6 m/s). h indicates prosthetic height.

Three prosthetic manufacturers, Össur (Reykjavik, Iceland), Freedom Innovations (Irvine, CA, USA), and Ottobock (Duderstadt, Germany) donated a combined total of 55 RSPs for use in our study. We characterized prosthetic stiffness profiles and hysteresis magnitudes from 14 C-shaped Össur Flex-Run prostheses (stiffness categories 3 low– 7 high), 12 C-shaped Freedom Innovations Catapult FX6 prostheses (stiffness categories 2–7), 14 J-shaped Ottobock 1E90 Sprinter prostheses (stiffness categories 1–5), and 15 J-shaped Össur Cheetah Xtend prostheses (stiffness categories 2–7) (Fig 2) (Table 1). The unique design of the Catapult prosthesis allows for stiffness modifications via interchangeable carbon-fiber supports (PowerSprings) that are designed to supplement overall stiffness [20] (Fig 2). PowerSprings have designated stiffness categories based on the manufacturer’s categorization. We tested each Catapult with the PowerSpring of the matching stiffness category (e.g. a category 2 Catapult with a category 2 PowerSpring).

**Table 1:** The manufacturer recommended running-specific prosthesis (RSP) stiffness categories with the corresponding body mass for distance running and sprinting, plus the quantity of RSPs tested.

RSP Model	Stiffness Category	Body Mass (kg)		Quantity of RSPs
		Distance Running	Sprinting	
Össur Flex-Run	3 Low	53–56	N/A	1
	3 High	56–59		1
	4 Low	60–64.5		1
	4 High	64.5–68		2
	5 Low	69–73		1
	5 High	73–77		2
	6 Low	78–83		1
	6 High	83–88		2
	7 Low	89–94.5		1
	7 High	94.5–100		2
Freedom Innovations Catapult FX6	2	53–59	N/A	2
	3	60–68		2
	4	69–77		2
	5	78–88		2
	6	89–100		2
	7	101–116		2
Ottobock 1E90 Sprinter	1	40–59	40–52	3
	2	60–70	53–63	3
	3	71–86	64–79	3
	4	87–102	80–95	3
	5	103–118	96–111	3
Össur Cheetah Xtend	2	53–59	53–59	2
	3	60–68	60–68	2
	4	69–77	69–77	3
	5	78–88	78–88	3
	6	89–100	89–100	3
	7	101–116	101–116	2

### Stiffness Testing

To assess prosthetic stiffness and hysteresis at conditions that matched those of our analyzed running data, we fabricated an aluminum attachment to secure the RSPs on to the force transducer of our materials testing machine (Instron Series 5859, Norwood, MA, USA) (Fig 2). We also constructed an aluminum rotating base and fixed it under each C-shaped RSP at 0°, α_3_, and α_6_, as well as under each J-shaped RSP at 0°, β_3_, and β_6_ (Fig 2). We applied three successive loading and unloading cycles at 100 N/s on each RSP for each condition. This loading rate was relatively fast and ensured that our materials testing machine operated within the safe speed range, even with our most compliant RSPs. Three compressive loading and unloading cycles matched the number of cycles from Brüggeman et al. [8].

To determine the peak GRF magnitude applied on each RSP, we considered the heaviest manufacturer recommended body weight for each prosthetic stiffness category, then multiplied it by 3.0 to replicate the upper limit of peak GRFs typically produced by affected legs while running 3 m/s [16], and by 3.5 to replicate the upper limit of peak GRFs produced by affected legs while running 6 m/s [16]. We compared the effects of testing angle and prosthetic height on stiffness and hysteresis by evaluating prosthetic compression with an applied peak resultant GRF of 3.0 times the largest recommended body weight for each RSP. We minimized shearing forces by using a low-friction roller-system beneath each RSP that allowed anterior and posterior translation while maintaining the angle of the applied force relative to the longitudinal axis (Fig 2) [30]. We set the threshold for force detection at 10 N. We recorded applied force magnitudes and prosthetic displacement measurements at 10 Hz, which, when combined with the loading rate (100 N/s), allowed the measurement of force-displacement data from every 10 N of applied force; ~150 to 400 data points per loading cycle.

To determine the effect of prosthetic height on the stiffness of C-shaped RSPs, we tested the Catapult and Flex-Run prostheses at 38.2 cm and 69.7 cm by altering the aluminum pylon height. To determine the effect of height on the stiffness of J-shaped RSPs, we tested the 1E90 Sprinter prostheses at 25.0, 31.5, and 38.0 cm, and the Cheetah Xtend prostheses at 31.5, 38.0, and 41.5 cm. Prosthetic height was measured vertically from the ground to the base of our height adjustment attachment in an unloaded state (Fig 2). We chose to test C-shaped RSPs across the largest possible height range given our components. We tested J-shaped RSPs at heights that spanned the largest possible range while allowing matched height comparisons (31.5 cm and 38.0 cm) between different models.

### Analyses

To characterize prosthetic stiffness, we calculated the average coefficients of determination (R^2^) for linear and curvilinear characterizations of the applied force relative to the vertical displacement for each 3-cycle trial. Next, we averaged R^2^ values within and across trials for a given prosthetic model, stiffness category, height, and testing angle combination. Furthermore, we calculated average prosthetic stiffness for each model across stiffness categories using the force-displacement function during simulated running conditions.

For every cycle, we calculated hysteresis as the ratio of energy lost during recoil relative to the energy stored during compression, then expressed it as a percentage:

$$Hysteresis=\frac{\int_{o}^{H}F(h)dh-\int_{H}^{o}F(h)dh}{\int_{o}^{H}F(h)dh}\times 100$$ (2)

where *F* is the applied force as a function of the change in prosthetic height (*h*) and peak change in prosthetic height (*H*) of the corresponding cycle. Hysteresis was averaged for each 3-cycle trial, and averaged across trials of the same prosthetic model, stiffness category, height, and testing angle. We measured prosthetic stiffness and hysteresis with the respective manufacturers supplied rubber sole. We also measured the stiffness and hysteresis of the highest stiffness category from each model at 0° without the rubber sole.

### Statistical Analyses

We used paired two-tailed t-tests to compare average R^2^ values from linear and curvilinear force-displacement functions across prosthetic models and to compare the manufacturer recommended stiffness across prosthetic models for athletes at body masses of 55 kg to 100 kg in 5 kg increments using the average angles and peak applied force magnitudes produced at 3 m/s (α_3_ and β_3_) from the C- and J-shaped RSPs, respectively. We also used paired two-tailed t-tests to compare the prescribed stiffness of different prosthetic models for athletes at body masses of 55 kg to 100 kg in 5 kg increments using the average angles and peak applied force magnitudes produced at 6 m/s (α_6_ and β_6_) from the C- and J-shaped RSPs, respectively. The recommended stiffness values for J-shaped RSPs were calculated using the tallest mutual height (38 cm).

Moreover, for C-shaped RSPs, we used linear mixed models to compare 1) prosthetic stiffness and 2) hysteresis for each prosthetic model across stiffness categories, testing angles, and interaction effects. For the J-shaped RSPs we included prosthetic height as an independent variable and used two linear mixed models to compare 1) prosthetic stiffness and 2) hysteresis for each prosthetic model across stiffness categories, testing angles, and heights, in addition to their interactions. We performed paired two-tailed t-tests to assess the influence of the prosthetic sole on stiffness and hysteresis. We carried out our statistical analyses using R-studio (Boston, MA, USA) software. Significance was set at p<0.05. When applicable, we implemented the Bonferroni correction to account for multiple comparisons.

## Results

### Subject Data

When participants used C-shaped RSPs to run 3 m/s, the average angle of their RSP’s longitudinal axis relative to the peak resultant GRF was 15.1° ± 4.8° and the mean peak resultant GRF was 2.5 ± 0.3 times body weight. At 6 m/s the average angle was 10.0° ± 4.2° and the peak resultant GRF was 2.7 ± 0.3 times body weight. When participants used a J-shaped RSP to run 3 m/s, the average angle of their RSP’s longitudinal axis relative to the peak resultant GRF was 20.9° ± 8.9° while the average peak resultant GRF was 2.6 ± 0.3 times body weight. At 6 m/s, the average angle was 24.2° ± 9.3° and the average peak resultant GRF magnitude was 2.8 ± 0.3 times body weight. Since our custom base was constructed to rotate in incremental steps, we used the following values for RSP testing: α_3_ = 15.0°, α_6_ = 10.0°, β_3_ = 20.0°, and β_6_ = 25.0°.

### Prosthetic force-displacement characteristics

**Fig 3:**
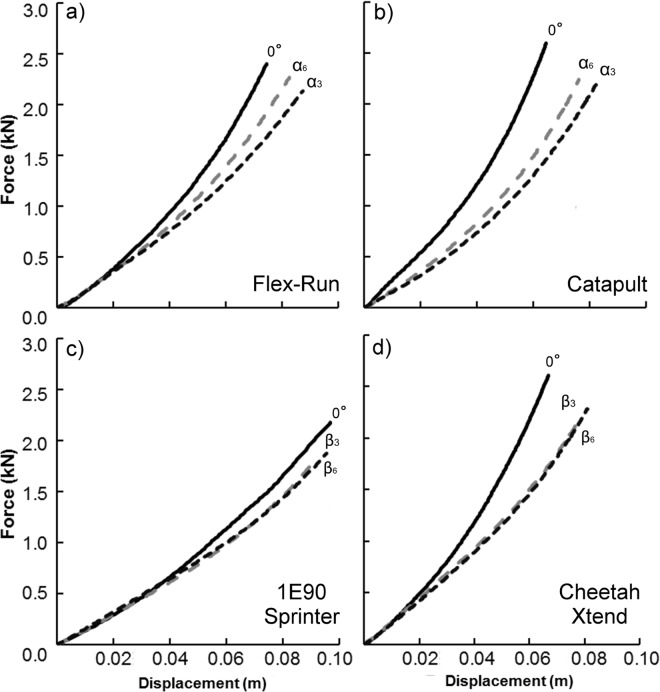
Representative force-displacement profiles for running-specific prosthetic models at each testing angle. Each running-specific prosthesis (RSP) is the manufacturer recommended stiffness category for a 70 kg distance runner. α_3_ and β_3_ indicate the measured angle between the RSP and peak resultant ground reaction force (GRF) vector while running 3 m/s using the C-shaped RSPs (Flex-Run and Catapult) and J-shaped RSPs (1E90 Sprinter and Cheetah Xtend), respectively. α_6_ and β_6_ indicate the measured angles between the RSP and peak resultant GRF vector while running 6 m/s using the C-shaped RSPs and J-shaped RSPs, respectively. a) The Flex-Run prosthesis at testing angles of 0°, α_3_, and α_6,_ b) the Catapult prosthesis at testing angles of 0°, α_3_, and α_6,_ c) the 1E90 Sprinter prosthesis at testing angles of 0°, β_3_, and β_6_, and d) the Cheetah Xtend prosthesis at testing angles of 0°, β_3_, and β_6._

Overall, characterizing the slope of the force-displacement curves with a 2^nd^ degree polynomial explained 4.4% more of the variance than a linear function using angles indicative of 3 m/s and 6 m/s (p<0.001) (Fig 3). At a testing angle of 0°, a 2^nd^ degree polynomial explained 5.0% more of the variance than using a linear function (p<0.001). We did not explore functions beyond a 2^nd^ degree polynomial due to its impeccable fit (average R^2^ = 0.998).

### Prosthetic Prescription

**Fig 4:**
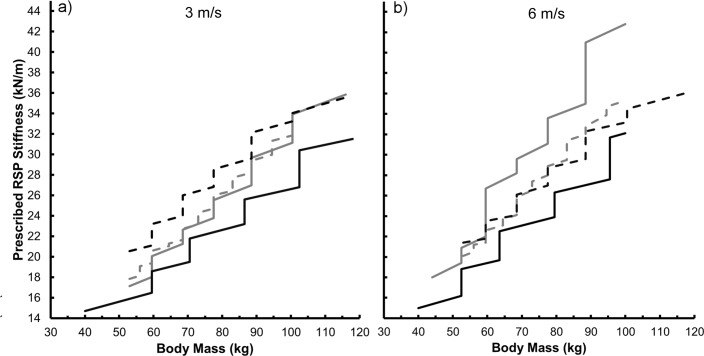
Prescribed prosthetic stiffness. The average stiffness (kN/m) of each running-specific prosthesis (RSP) as a function of the respective manufacturer’s recommended user body mass (kg) at running speeds of 3 m/s (a), and 6 m/s (b). The stiffness of each RSP was calculated using peak applied force magnitudes that simulated running 3 m/s (α_3_ and β_3_) and 6 m/s (α_6_ and β_6_). We then calculated displacement using the mean curvilinear force-displacement profiles with the appropriate applied force magnitudes. See S1–S4 Tables.

Using the peak resultant GRFs and angles produced at 3 m/s, the actual stiffness of the manufacturer recommended Cheetah Xtend, which is prescribed based on user body mass, was 4% to 15% stiffer than the Flex-Run (p<0.001), 7% to 19% stiffer than the Catapult (p<0.001), and 20% to 28% stiffer than the 1E90 Sprinter (p<0.001) prostheses across matched user body masses (Fig 4). Using the peak resultant GRFs and angles produced at 6 m/s, the manufacturer recommended Cheetah Xtend prostheses were the same stiffness as the Flex-Run (p = 0.166), 0% to 22% less stiff than the Catapult (p = 0.001), and 3% to 21% stiffer than the 1E90 Sprinter (p<0.001) prostheses at matched user body masses (Fig 4). The Flex-Run and Catapult prostheses are not specifically recommended for fast running/sprinting; therefore we used manufacturer recommended stiffness categories for distance running at 6 m/s.

Prosthetic stiffness depends on peak GRF magnitude; hence we calculated the average 2^nd^ order polynomial equations for each prosthetic model and stiffness category (S1-S4) so that prosthetists can predict an athlete’s prosthetic stiffness from the amount of force they apply on the ground and/or prosthetic compression. For those unable to quantify force magnitudes or compression, and because of the relatively linear force-displacement relationships (average R^2^ = 0.956), we also report average linear stiffness values (Table 2).

**Table 2:** The manufacturer recommended average prosthetic stiffness across models based on running 3 m/s and 6 m/s. All values include the rubber sole that comes with the prosthetic model, with the exception of the Össur Cheetah Xtend, which was equipped with the Össur Flex-Run’s rubber sole.

Users Mass (kg)	3 m/s				6 m/s			
	Flex-Run (kN/m)	Catapult (kN/m)	1E90 Sprinter (kN/m)	Cheetah Xtend (kN/m)	Flex-Run (kN/m)	Catapult (kN/m)	1E90 Sprinter (kN/m)	Cheetah Xtend (kN/m)
**55**	18.0	17.4	16.2	20.7	20.4	20.4	19.0	21.5
**60**	20.6	20.1	18.6	23.2	22.6	25.8	19.5	23.5
**65**	22.1	20.8	19.1	23.7	23.7	27.6	22.7	23.9
**70**	22.9	22.8	21.8	26.1	26.1	29.9	23.1	26.4
**75**	23.7	23.5	22.2	26.6	27.7	30.7	23.5	26.8
**80**	26.2	25.9	22.7	28.8	29.2	33.7	26.4	28.9
**85**	26.1	26.5	23.2	29.3	31.3	34.5	26.8	29.4
**90**	29.5	29.9	25.9	32.3	33.4	41.2	27.2	32.4
**95**	31.4	30.5	26.3	32.7	34.7	42.0	27.6	32.8
**100**	31.8	31.1	26.7	33.2	35.3	42.8	32.1	33.1

### Hysteresis

The percentage of mechanical energy lost per cycle for C-shaped RSPs across conditions averaged 5.14% (SD: 0.70%). For every 1° increase in testing angle, the hysteresis magnitude decreased 0.04% (p<0.001). The average hysteresis for J-shaped RSPs across conditions was 4.28% (SD: 0.65%), which was lower than that of the C-shaped RSPs (p<0.001). Furthermore, testing angle affected the hysteresis of J-shaped RSPs (p<0.001), while height had no effect (p = 0.215). For every 1° increase in testing angle, the hysteresis of the 1E90 Sprinter and Cheetah Xtend prostheses decreased 0.01% and 0.08%, respectively (p<0.001). Additionally, removing the rubber soles from C- and J-shaped RSPs reduced the hysteresis magnitudes by 42% (p<0.001).

### Effect of angle and height on prosthetic stiffness

While controlling for prosthetic height, every 1° increase in testing angle decreased the stiffness of the Flex-Run and Catapult prostheses by 0.41 kN/m (p<0.001) and 0.79 kN/m (p<0.001), respectively (Fig 3). Every 1° increase in testing angle decreased the stiffness of the 1E90 Sprinter and Cheetah Xtend prostheses by 0.45 kN/m (p<0.001) and 0.76 kN/m (p<0.001), respectively. Moreover, at a fixed testing angle, every 1 cm increase in height decreased the stiffness of both J-shaped RSPs by 0.27 kN/m (p<0.001). Despite a drastic pylon height difference (31.5 cm), preliminary testing revealed no effect of height on the stiffness of C-shaped RSPs; therefore we did not further test the effect of height across C-shaped RSPs. Furthermore, removing the rubber soles did not affect prosthetic stiffness across models (p = 0.151).

## Discussion

Despite well-characterizing the force-displacement relationships of the RSPs (average R^2^ = 0.956), a linear function did not fit quite as well as a 2^nd^ degree polynomial function (p<0.001), leading us to partially reject our initial hypothesis. Contrary to the notion that prosthetic stiffness is invariant during running [8,16–18], our data suggest that as athletes exert greater forces on the ground and/or adjust the angle between the peak resultant GRF and their RSP during stance, prosthetic stiffness is altered. For example, a 70 kg athlete that produces peak resultant GRFs of 2.2, 2.6, 3.0, 3.4 times body weight with their affected leg using a manufacturer recommended Cheetah Xtend prosthesis (height: 38 cm; angle: 25.0°) would exhibit stiffness values of 25.1, 26.1, 27.1, and 28.1 kN/m, respectively. Yet, if the 70 kg athlete increased the angle of their RSP with respect to the resultant GRF from 15° to 30° in 5° increments, the aforementioned prosthetic stiffness values would change to 32.7, 29.9, 27.1, 24.3 kN/m. It is possible that the inverse relationship between affected leg stiffness and running speed found in McGowan et al. [16] can be attributed to decreased prosthetic stiffness via increased angles between the resultant GRF vectors and RSPs at faster speeds.

Overall, mechanically testing RSPs at 0° overestimates prosthetic stiffness (linear) by 10% to 39% compared to using angles utilized by athletes with transtibial amputations while running at 3 m/s and 6 m/s. Previous studies have tested the stiffness of RSPs at 0° [8], and 30° [19]. We compared our methodology to that of Brüggeman et al. [8] by acquiring the same prosthetic model (Össur Cheetah) as the previous study, replicating their protocol (applied force: 1500 N, testing angle: 0°, loading velocity: 1 m/min), and then using our method (applied force: 2724 N, testing angle: 25°, loading velocity: 100 N/s) to determine stiffness. Brüggeman et al.’s protocol resulted in a prosthetic stiffness (linear) of 34.2 kN/m, whereas our protocol resulted in a linear stiffness of 29.2 kN/m. These discrepancies suggest that prosthetic stiffness testing procedures should be standardized.

We reject our second hypothesis; manufacturer recommended prosthetic stiffness varies across models for a given user body mass and activity. Additionally, we compared manufacturer recommended prosthetic stiffness during running at 6 m/s versus at 3 m/s. At a given body mass (prosthetic height of 38 cm), the manufacturer recommended 1E90 Sprinter prostheses were 11% stiffer at 6 m/s compared to 3 m/s across a 45 kg span in user body mass (p = 0.003). Also, the recommended Catapult prosthetic stiffness increased 32% due to a greater recommended prosthetic stiffness category and reduced angle between the RSP and peak resultant GRF (Fig 4). Conversely, the Cheetah Xtend prostheses are recommended at the same stiffness categories for 3 m/s and 6 m/s [24], and thus the stiffness values varied by <1% (Bonferroni corrected p-value: p = 0.080). Prosthetic stiffness requirements may be different for running at various speeds due to the different mechanical demands of the respective tasks. Future studies are needed to assess the effects of prosthetic stiffness on distance running and sprinting performance.

Since testing angle affected hysteresis, we also reject our third hypothesis stating that prosthetic hysteresis would be invariant across testing conditions. Intriguingly, RSPs dissipate less energy when their proximal end is rotated forward with respect to the applied force. Future studies are needed to examine prosthetic designs and decipher why RSPs display less hysteresis when rotated forward. Due to the importance of mechanical energy return on running and sprinting performance [4,5], the designs of future RSPs should be developed to mitigate mechanical energy dissipation.

Moreover, prosthetic hysteresis was 42% lower when we removed the rubber soles, indicating that the rubber soles were responsible for almost half of the dissipated energy. Athletes with leg amputations should use soles with minimal damping to maximize the mechanical energy return of RSPs. In addition to the sole, energy dissipation probably occurs at the residual limb/socket interface. To our knowledge, no study has quantified the mechanical behavior of the residual limb and socket interface while running. Improving socket design by enhancing the connection between athletes and their RSPs may allow better utilization of the returned mechanical energy and potentially improve running performance.

Pylon height does not affect the stiffness of C-shaped RSPs; therefore, we reject our final hypothesis. The aluminum pylon of C-shaped RSPs has an annular section (i.e. an empty cylinder) and appears less prone to bending due to the perpendicular components of the applied compression forces, and due to a higher area moment of inertia [31] compared to the rectangular section of J-shaped RSPs. Increasing the overall length of the aluminum pylon technically reduces its overall stiffness, but the lengths used in our measurements were not enough to elicit a measurable difference. The height of RSPs needs to be within a relatively narrow range for athletes with unilateral amputations due to their unaffected leg length. Therefore prosthetic stiffness adjustments would primarily be accomplished by changing stiffness category or sagittal plane angle. On the other hand, athletes with bilateral amputations can consider a wide range of heights and stiffness categories to achieve a specified prosthetic stiffness; however, height and stiffness may affect running performance in different ways. In addition to stiffness, the effects of prosthetic height and alignment on performance warrant future research.

We assumed that the C-shaped RSPs were perpendicular to the respective pylons. Yet, the sagittal plane RSP-pylon alignment may have been slightly altered due to individual preference, thus our reported angles between the C-shaped RSPs and resultant GRF vectors may have been over/underestimated by a few degrees. We collected prosthetic angles and peak resultant GRFs from a cohort of exceptional athletes with unilateral transtibial amputations at 3 m/s and 6 m/s. Conceivably, less athletic individuals with amputations, or athletes with different amputation levels may not utilize the same prosthetic angles and/or generate the same resultant GRFs compared to those exhibited by our participants, and consequently prosthetic stiffness may differ. For example, athletes with transfemoral amputations with pylons connecting their RSPs to their sockets can use our reported values at 0°, as it is a fair approximation of their RSP-peak GRF angle to determine the prosthetic stiffness and hysteresis.

Our methodology does not account for the rotation of the RSP with respect to the resultant GRF throughout ground contact. It may be that RSPs are stiffer at initial and terminal ground contact than at mid-stance due to a smaller angle between the RSP and resultant GRF vector. On the other hand, as applied force accrues RSPs become stiffer, implying that RSPs are stiffest at mid-stance. The influence of angle and force may counteract each other, exhibiting a constant prosthetic stiffness throughout stance; perhaps a deliberate design choice of prosthetic manufacturers. Future studies are warranted to include a rotational component to the mechanical stiffness testing of RSPs. Furthermore, we tested our RSPs with a loading rate (100 N/s) that is much lower than that recorded during running (over 4000 N/s [16,18]). However, our low loading rate (100 N/s) enabled us to record force-displacement data from every 10 N of applied force, thus presenting ~150 to 400 data points per loading cycle. When athletes with an amputation run 6 m/s, they have a ground contact time of ~0.2 seconds [18,25]. If ground reaction forces were recorded at 2000 Hz, then 200 data points would have been collected from initial ground contact to mid-stance/peak GRF, which coincides with our material testing machines sampling versus loading rate data. Nevertheless, it is ideal for prosthetic testing to mimic the loading/unloading rates of those recorded during running; unfortunately these rates are beyond the capability of our equipment.

## Conclusions

We assessed prosthetic stiffness and hysteresis across a wide range of models, stiffness categories, and heights, at forces and angles that simulate those exhibited by athletes with transtibial amputations running at 3 m/s and 6 m/s. We found that the force-displacement profiles of RSPs are curvilinear, indicating that prosthetic stiffness varies with the magnitude of applied force. Yet, a linear force-displacement characterization is strongly predictive. We also found that manufacturer recommended prosthetic stiffness varies between models, and that the height of J-shaped RSPs is inversely related to stiffness. Moreover, we provide evidence that prosthetic stiffness is much greater at 0° than at angles representative of those that occur during running.

When athletes with leg amputations change prosthetic models, height, and/or sagittal plane alignment, prosthetic stiffness also changes; therefore variations in comfort, performance, etc. may be indirectly due to altered stiffness. We propose that prosthetic stiffness should be assessed under conditions that simulate the demands of the respective activity, and that manufacturers should provide the stiffness values of each RSP at specific heights. Until then, our study provides reference for the stiffness values of various prosthetic models across multiple stiffness categories and heights, and provides a foundation for future research to understand the potential effects of prosthetic stiffness on performance during distance running and sprinting.

## Supporting Information

S1 Table The stiffness and hysteresis characteristics for Össur Flex-Run prostheses at each testing condition. The equations indicate prosthetic displacement in meters (h) used to calculate the applied force in kN. Stiffness equals applied force divided by displacement. a and b are constants. All prostheses were tested with the manufacturer supplied sole, with the exception of stiffness category 7 High No Sole.
(DOCX)

S2 Table The stiffness and hysteresis characteristics for Freedom Innovations Catapult FX6 prostheses at each testing condition. The equations indicate prosthetic displacement in meters (h) used to calculate the applied force in kN. Stiffness equals applied force divided by displacement. a and b are constants. All prostheses were tested with the manufacturer supplied sole, with the exception of stiffness category 7 No Sole.
(DOCX)

S3 Table The stiffness and hysteresis characteristics for Ottobock 1E90 Sprinter prostheses at each testing condition. The equations indicate prosthetic displacement in meters (h) used to calculate the applied force in kN. Stiffness equals applied force divided by displacement. a and b are constants. All prostheses were tested with the manufacturer supplied sole, with the exception of stiffness category 5 No Sole.
(DOCX)

S4 Table The stiffness and hysteresis characteristics for the Össur Cheetah Xtend prostheses at each testing condition. The equations indicate prosthetic displacement in meters (h) used to calculate the applied force in kN. Stiffness equals applied force divided by displacement. a and b are constants. All RSPs were tested with the supplied sole from the Össur Flex-Run prostheses, with the exception of stiffness category 7 No Sole.
(DOCX)
