# Correction: Characterizing the Mechanical Properties of Running-Specific Prostheses

**DOI:** 10.1371/journal.pone.0173764

**Published:** 2017-03-13

**Authors:** Owen N. Beck, Paolo Taboga, Alena M. Grabowski

The Supporting Information files are incorrectly labeled and described. Please see the correct labels and descriptions for the Supporting Information files here:

The proper caption for S1 Table is: The stiffness and hysteresis characteristics for Freedom Innovations Catapult FX6 prostheses at each testing condition.

The proper caption for S2 Table is: The stiffness and hysteresis characteristics for Össur Flex-Run prostheses at each testing condition.

The proper caption for S3 Table is: The stiffness and hysteresis characteristics for the Össur Cheetah Xtend prostheses at each testing condition.

The proper caption for S4 Table is: The stiffness and hysteresis characteristics of Ottobock 1E90 Sprinter prostheses at each testing condition.

## Supporting information

S1 Table The stiffness and hysteresis characteristics for Freedom Innovations Catapult FX6 prostheses at each testing condition. The equations indicate prosthetic displacement in meters (h) used to calculate the applied force in kN. Stiffness equals applied force divided by displacement. a and b are constants. All prostheses were tested with the manufacturer supplied sole, with the exception of stiffness category 7 No Sole.
(DOCX)

S2 Table The stiffness and hysteresis characteristics for Össur Flex-Run prostheses at each testing condition. The equations indicate prosthetic displacement in meters (h) used to calculate the applied force in kN. Stiffness equals applied force divided by displacement. a and b are constants. All prostheses were tested with the manufacturer supplied sole, with the exception of stiffness category 7 High No Sole.
(DOCX)

S3 Table The stiffness and hysteresis characteristics for the Össur Cheetah Xtend prostheses at each testing condition. The equations indicate prosthetic displacement in meters (h) used to calculate the applied force in kN. Stiffness equals applied force divided by displacement. a and b are constants. All RSPs were tested with the supplied sole from the Össur Flex-Run prostheses, with the exception of stiffness category 7 No Sole.
(DOCX)

S4 Table The stiffness and hysteresis characteristics of Ottobock 1E90 Sprinter prostheses at each testing condition. The equations indicate prosthetic displacement in meters (h) used to calculate the applied force in kN. Stiffness equals applied force divided by displacement. a and b are constants. All prostheses were tested with the manufacturer supplied sole, with the exception of stiffness category 5 No Sole.
(DOCX)
