# Supplementary material for: Characterizing the Mechanical Properties of Running-Specific Prostheses
Source: PLoS One. 2016 Dec 14;11(12):e0168298. doi: 10.1371/journal.pone.0168298 (PMC5156386; doi:10.1371/journal.pone.0168298)
Supplement: S2 Table — The equations indicate prosthetic displacement in meters (h) used to calculate the applied force in kN. Stiffness equals applied force divided by displacement. a and b are constants. All prostheses were tested with the manufacturer supplied sole, with the exception of stiffness category 7 No Sole. (DOCX) [file pone.0168298.s002.docx]

**Supplementary Material Table 1**

| **Össur Flex-Run** | | | | |
| --- | --- | --- | --- | --- |
| **Condition**  **(Angle)** | **Stiffness**  **Category** | **Force=ah^2^+bh** | **Stiffness**  **Variability**  **(SD)** | **Percent**  **Hysteresis**  **Mean (SD)** |
|  | 3 Low | 195h^2^+10h | - | 5.0 |
|  | 3 High | 199h^2^+12h | - | 5.4 |
| Neutral  (0°) | 4 Low | 187h^2^+12h | - | 5.5 |
|  | 4 High | 223h^2^+12h | (1.3) | 5.7 (0.2) |
|  | 5 Low | 269h^2^+12h | - | 5.7 |
|  | 5 High | 271h^2^+14h | (2.8) | 6.2 (0.6) |
|  | 6 Low | 270h^2^+15h | - | 5.4 |
|  | 6 High | 298h^2^+17h | (0.7) | 5.3 (0.2) |
|  | 7 Low | 336h^2^+18h | - | 4.9 |
|  | 7 High | 354h^2^+18h | (0.3) | 5.7 (0.1) |
|  | 7 High No Sole | 327h^2^+21h | - | 2.8 |
|  | 3 Low | 102h^2^+10h | - | 4.7 |
|  | 3 High | 101h^2^+12h | - | 4.7 |
| 3 m/s  (15°) | 4 Low | 102h^2^+13h | - | 4.4 |
|  | 4 High | 109h^2^+13h | (0.3) | 4.7 (0.3) |
|  | 5 Low | 121h^2^+14h | - | 6.8 |
|  | 5 High | 122h^2^+15h | (0.6) | 4.8 (0.0) |
|  | 6 Low | 122h^2^+17h | - | 4.4 |
|  | 6 High | 137h^2^+18h | (0.4) | 4.6 (0.1) |
|  | 7 Low | 161h^2^+18h | - | 4.5 |
|  | 7 High | 165h^2^+19h | (0.7) | 4.7 (0.1) |
|  | 3 Low | 134h^2^+11h | - | 5.8 |
|  | 3 High | 133h^2^+12h | - | 4.9 |
|  | 4 Low | 133h^2^+13h | - | 4.4 |
| 6 m/s  (10°) | 4 High | 147h^2^+13h | (0.1) | 4.8 (0.5) |
|  | 5 Low | 175h^2^+14h | - | 4.9 |
|  | 5 High | 174h^2^+15h | (0.4) | 5.0 (0.3) |
|  | 6 Low | 180h^2^+16h | - | 4.6 |
|  | 6 High | 206h^2^+17h | (0.5) | 4.7 (0.2) |
|  | 7 Low | 232h^2^+17h | - | 4.6 |
|  | 7 High | 223h^2^+19h | (0.0) | 5.0 (0.1) |
