# Supplementary material for: Characterizing the Mechanical Properties of Running-Specific Prostheses
Source: PLoS One. 2016 Dec 14;11(12):e0168298. doi: 10.1371/journal.pone.0168298 (PMC5156386; doi:10.1371/journal.pone.0168298)
Supplement: S3 Table — The equations indicate prosthetic displacement in meters (h) used to calculate the applied force in kN. Stiffness equals applied force divided by displacement. a and b are constants. All prostheses were tested with the manufacturer supplied sole, with the exception of stiffness category 5 No Sole. (DOCX) [file pone.0168298.s003.docx]

**Supplementary Material Table 3.**

| **Össur Cheetah Xtend** | | | | | | | | | | |
| --- | --- | --- | --- | --- | --- | --- | --- | --- | --- | --- |
| **Condition**  **(Angle)** | **Stiffness**  **Category** | **Force=ah^2^+bh** | | | **Stiffness**  **Variability (SD)** | | | **Percent Hysteresis**  **Mean (SD)** | | |
|  |  | 31.5 cm | 38.0 cm | 44.5 cm | 31.5 cm | 38.0 cm | 44.5 cm | 31.5 cm | 38.0 cm | 44.5 cm |
| Neutral  (0°) | 2 | 262h^2^+16h | 253h^2^+14h | 213h^2^+15h | (0.4) | (1.2) | - | 5.1 (0.7) | 5.1 (0.7) | 5.0 |
|  | 3 | 306h^2^+16h | 322h^2^+15h | 291h^2^+16h | (0.4) | (0.2) | (0.0) | 7.5 (0.1) | 7.5 (0.1) | 5.6 (0.0) |
|  | 4 | 345h^2^+16h | 348h^2^+16h | 304h^2^+15h | (0.9) | (0.4) | (0.9) | 7.1 (1.5) | 7.1 (1.5) | 6.2 (0.8) |
|  | 5 | 387h^2^+17h | 359h^2^+18h | 321h^2^+17h | (1.3) | (0.6) | (0.3) | 7.6 (0.3) | 7.6 (0.3) | 7.0 (0.3) |
|  | 6 | 420h^2^+18h | 404h^2^+19h | 331h^2^+18h | (1.6) | (1.3) | (1.6) | 8.1 (1.0) | 8.1 (1.0) | 6.5 (0.8) |
|  | 7 | 440h^2^+21h | 429h^2^+20h | 344h^2^+21h | (0.7) | (0.4) | (0.7) | 7.3 (0.5) | 7.3 (0.5) | 6.6 (1.2) |
|  | 7 No Sole | **-** | **-** | 484h^2^+16h | - | - | - | **-** | **-** | 3.0 |
| 3 m/s  (20°) | 2 | 99h^2^+15h | 95h^2^+14h | 94h^2^+14h | (0.3) | (0.3) | - | 4.4 (0.1) | 4.4 (0.1) | 4.3 |
|  | 3 | 108h^2^+17h | 111h^2^+16h | 114h^2^+15h | (0.0) | (0.3) | (0.6) | 4.9 (0.3) | 4.9 (0.3) | 4.8 (0.5) |
|  | 4 | 130h^2^+18h | 131h^2^+17h | 130h^2^+17h | (0.5) | (0.4) | (0.1) | 4.5 (0.4) | 4.5 (0.4) | 4.7 (0.3) |
|  | 5 | 158h^2^+18h | 146h^2^+19h | 149h^2^+18h | (0.6) | (0.2) | (0.2) | 5.4 (1.4) | 5.4 (1.4) | 4.5 (0.5) |
|  | 6 | 177h^2^+20h | 167h^2^+20h | 167h^2^+20h | (0.1) | (0.1) | (0.1) | 5.5 (0.2) | 5.5 (0.2) | 4.8 (0.3) |
|  | 7 | 193h^2^+20h | 195h^2^+19h | 195h^2^+19h | (0.7) | (0.5) | (0.3) | 5.4 (0.2) | 5.4 (0.2) | 4.6 (0.4) |
| 6 m/s  (25°) | 2 | 74h^2^+16h | 68h^2^+17h | 68h^2^+17h | (1.0) | (0.0) | - | 4.3 (0.1) | 4.3 (0.1) | 3.8 |
|  | 3 | 90h^2^+17h | 82h^2^+18h | 82h^2^+18h | (1.0) | (0.3) | (0.6) | 4.8 (0.9) | 4.8 (0.9) | 4.0 (0.0) |
|  | 4 | 96h^2^+20h | 100h^2^+19h | 98h^2^+19h | (0.6) | (0.6) | (0.5) | 4.2 (0.3) | 4.2 (0.3) | 3.8 (0.0) |
|  | 5 | 121h^2^+21h | 116h^2^+20h | 115h^2^+20h | (0.1) | (0.2) | (0.4) | 4.4 (0.0) | 4.4 (0.0) | 3.7 (0.0) |
|  | 6 | 115h^2^+24h | 113h^2^+24h | 118h^2^+23h | (0.5) | (1.0) | (0.3) | 4.1 (0.0) | 4.1 (0.0) | 4.9 (0.4) |
|  | 7 | 154h^2^+22h | 159h^2^+22h | 149h^2^+22h | (0.1) | (0.0) | (0.8) | 5.3 (0.8) | 5.3 (0.8) | 4.3 (0.5) |
