# Supplementary material for: Correction: Characterizing the Mechanical Properties of Running-Specific Prostheses
Source: PLoS One. 2017 Mar 13;12(3):e0173764. doi: 10.1371/journal.pone.0173764 (PMC5348000; doi:10.1371/journal.pone.0173764)
Supplement: S1 Table — The equations indicate prosthetic displacement in meters (h) used to calculate the applied force in kN. Stiffness equals applied force divided by displacement. a and b are constants. All prostheses were tested with the manufacturer supplied sole, with the exception of stiffness category 7 No Sole. (DOCX) [file pone.0173764.s001.docx]

**S1 Table: The stiffness and hysteresis characteristics for Freedom Innovations Catapult FX6 prostheses at each testing condition.**

| **Freedom Innovations Catapult FX6** | | | | |
| --- | --- | --- | --- | --- |
| **Condition**  **(Angle)** | **Stiffness**  **Category** | **Force=ah^2^+bh** | **Stiffness**  **Variability (SD)** | **Percent**  **Hysteresis**  **Mean (SD)** |
| Neutral  (0°) | 2 | 297h^2^+8h | (1.4) | 6.5 (0.6) |
|  | 3 | 346h^2^+11h | (2.2) | 6.2 (0.2) |
|  | 4 | 359h^2^+15h | (2.9) | 5.8 (0.2) |
|  | 5 | 411h^2^+17h | (1.6) | 5.9 (0.1) |
|  | 6 | 381h^2^+25h | (1.8) | 6.1 (0.2) |
|  | 7 | 504h^2^+25h | (2.0) | 6.1 (0.2) |
|  | 7 No Sole | 553h^2^+43h | - | 3.2 |
|  | 2 | 176h^2^+4h | (0.2) | 5.6 (0.1) |
|  | 3 | 195h^2^+6h | (0.4) | 5.8 (0.0) |
| 3 m/s  (15°) | 4 | 200h^2^+8h | (2.2) | 5.7 (0.7) |
|  | 5 | 237h^2^+8h | (0.7) | 5.4 (0.2) |
|  | 6 | 247h^2^+12h | (0.2) | 5.5 (0.0) |
|  | 7 | 293h^2^+13h | (0.7) | 5.5 (0.1) |
| 6 m/s  (10°) | 2 | 195h^2^+5h | (1.5) | 5.7 (0.2) |
|  | 3 | 234h^2^+5h | (1.7) | 5.3 (0.2) |
|  | 4 | 307h^2^+9h | (1.9) | 5.4 (0.0) |
|  | 5 | 309h^2^+11h | (1.1) | 5.3 (0.1) |
|  | 6 | 318h^2^+14h | (0.5) | 5.3 (0.0) |
|  | 7 | 395h^2^+18h | (4.3) | 5.3 (0.0) |

The equations indicate prosthetic displacement in meters (h) used to calculate the applied force in kN. Stiffness equals applied force divided by displacement. a and b are constants. All prostheses were tested with the manufacturer supplied sole, with the exception of stiffness category 7 No Sole.
