# Supplementary material for: Correction: Characterizing the Mechanical Properties of Running-Specific Prostheses
Source: PLoS One. 2017 Mar 13;12(3):e0173764. doi: 10.1371/journal.pone.0173764 (PMC5348000; doi:10.1371/journal.pone.0173764)
Supplement: S4 Table — The equations indicate prosthetic displacement in meters (h) used to calculate the applied force in kN. Stiffness equals applied force divided by displacement. a and b are constants. All prostheses were tested with the manufacturer supplied sole, with the exception of stiffness category 5 No Sole. (DOCX) [file pone.0173764.s004.docx]

**S4 Table: The stiffness and hysteresis characteristics of Ottobock 1E90 Sprinter prostheses at each testing condition.**

| **Ottobock 1E90 Sprinter** | | | | | | | | | | |
| --- | --- | --- | --- | --- | --- | --- | --- | --- | --- | --- |
| **Condition**  **(Angle)** | **Stiffness**  **Category** | **Force=ah^2^+bh** | | | **Stiffness**  **Variability (SD)** | | | **Percent Hysteresis**  **Mean (SD)** | | |
|  |  | 25.0 cm | 31.5 cm | 38.0 cm | 25.0 cm | 31.5 cm | 38.0 cm | 25.0 cm | 31.5 cm | 38.0 cm |
| Neutral  (0°) | 1 | 147h^2^+11h | 132h^2^+11h | 102h^2^+11h | (0.7) | (0.0) | (0.8) | 3.6 (0.4) | 3.6 (0.4) | 3.7 (0.4) |
|  | 2 | 152h^2^+14h | 143h^2^+13h | 109h^2^+13h | (2.2) | (0.1) | (0.3) | 5.3 (1.1) | 5.3 (1.1) | 3.8 (0.0) |
|  | 3 | 189h^2^+16h | 169h^2^+15h | 119h^2^+14h | (0.7) | (0.8) | (1.5) | 4.1 (0.2) | 4.1 (0.2) | 4.4 (0.8) |
|  | 4 | 214h^2^+20h | 168h^2^+17h | 140h^2^+18h | (2.3) | (1.4) | (2.6) | 3.6 (0.1) | 3.6 (0.1) | 4.0 (0.5) |
|  | 5 | 234h^2^+22h | 196h^2^+22h | 126h^2^+21h | (0.0) | (1.9) | (0.9) | 4.4 (1.1) | 4.4 (1.1) | 4.4 (0.4) |
|  | 5 No Sole | **-** | **-** | 231h^2^+20h | - | - | - | **-** | **-** | 1.2 |
| 3 m/s  (20°) | 1 | 90h^2^+9h | 90h^2^+9h | 85h^2^+9h | (0.3) | (0.0) | (0.1) | 3.8 (0.1) | 3.8 (0.1) | 3.8 (0.0) |
|  | 2 | 102h^2^+12h | 97h^2^+12h | 94h^2^+11h | (0.2) | (0.3) | (0.3) | 3.6 (0.1) | 3.6 (0.1) | 3.7 (0.0) |
|  | 3 | 123h^2^+14h | 117h^2^+14h | 115h^2^+13h | (0.6) | (0.4) | (0.5) | 3.8 (0.1) | 3.8 (0.1) | 4.0 (0.1) |
|  | 4 | 139h^2^+17h | 149h^2^+16h | 122h^2^+16h | (0.9) | (2.6) | (0.5) | 4.0 (0.2) | 4.0 (0.2) | 4.1 (0.2) |
|  | 5 | 146h^2^+22h | 142h^2^+21h | 127h^2^+20h | (0.4) | (0.2) | (0.3) | 5.1 (1.4) | 5.1 (1.4) | 4.5 (0.2) |
| 6 m/s  (25°) | 1 | 78h^2^+10h | 79h^2^+10h | 77h^2^+9h | (0.4) | (0.5) | (0.7) | 3.7 (0.1) | 3.7 (0.1) | 3.7 (0.2) |
|  | 2 | 86h^2^+13h | 84h^2^+13h | 82h^2^+13h | (0.4) | (0.1) | (0.2) | 4.0 (0.4) | 4.0 (0.4) | 3.8 (0.1) |
|  | 3 | 103h^2^+15h | 97h^2^+15h | 98h^2^+15h | (0.9) | (0.7) | (0.7) | 3.9 (0.5) | 3.9 (0.5) | 3.6 (0.1) |
|  | 4 | 117h^2^+18h | 112h^2^+18h | 107h^2^+17h | (0.8) | (0.8) | (0.7) | 4.0 (0.5) | 4.0 (0.5) | 4.4 (1.0) |
|  | 5 | 122h^2^+24h | 133h^2^+23h | 117h^2^+22h | (0.8) | (1.6) | (0.5) | 4.0 (0.1) | 4.0 (0.1) | 4.1 (0.5) |

The equations indicate prosthetic displacement in meters (h) used to calculate the applied force in kN. Stiffness equals applied force divided by displacement. a and b are constants. All prostheses were tested with the manufacturer supplied sole, with the exception of stiffness category 5 No Sole.
